# Disrupted development of sensory systems and the cerebellum in a zebrafish *ebf3a* mutant

**DOI:** 10.1093/g3journal/jkaf115

**Published:** 2025-05-23

**Authors:** Nghi D P Dang, Alexia K Barcus, Claire L Conklin, Thinh Q Truong, Michael D Vivian, Jun Wang, Holly R Thomas, John M Parant, Nan Cher Yeo, Summer B Thyme

**Affiliations:** Department of Pharmacology and Toxicology, The University of Alabama at Birmingham Heersink School of Medicine, The University of Alabama at Birmingham, Birmingham, AL 35294, USA; Department of Biochemistry and Molecular Biotechnology, University of Massachusetts Chan Medical School, University of Massachusetts, Worcester, MA 01605, USA; Department of Neurobiology, The University of Alabama at Birmingham Heersink School of Medicine, The University of Alabama at Birmingham, Birmingham, AL 35294, USA; Department of Pharmacology and Toxicology, The University of Alabama at Birmingham Heersink School of Medicine, The University of Alabama at Birmingham, Birmingham, AL 35294, USA; Department of Neurobiology, The University of Alabama at Birmingham Heersink School of Medicine, The University of Alabama at Birmingham, Birmingham, AL 35294, USA; Department of Pharmacology and Toxicology, The University of Alabama at Birmingham Heersink School of Medicine, The University of Alabama at Birmingham, Birmingham, AL 35294, USA; Department of Pharmacology and Toxicology, The University of Alabama at Birmingham Heersink School of Medicine, The University of Alabama at Birmingham, Birmingham, AL 35294, USA; Department of Pharmacology and Toxicology, The University of Alabama at Birmingham Heersink School of Medicine, The University of Alabama at Birmingham, Birmingham, AL 35294, USA; Department of Pharmacology and Toxicology, The University of Alabama at Birmingham Heersink School of Medicine, The University of Alabama at Birmingham, Birmingham, AL 35294, USA; Department of Biochemistry and Molecular Biotechnology, University of Massachusetts Chan Medical School, University of Massachusetts, Worcester, MA 01605, USA; Department of Neurobiology, The University of Alabama at Birmingham Heersink School of Medicine, The University of Alabama at Birmingham, Birmingham, AL 35294, USA

**Keywords:** zebrafish, neurodevelopment, autism spectrum disorder, sensory responsiveness

## Abstract

Mutations in the transcription factor early B cell factor 3 result in a neurodevelopmental disorder, and studies in animal models indicate that it has a critical role in neuronal differentiation. The molecular pathways and neuron types disrupted by its loss, however, have not been thoroughly investigated. Nor have the outcomes of these changes on behavior and brain activity. Here, we generated and characterized a zebrafish *ebf3a* loss-of-function mutant. We discovered morphological and neural phenotypes, including an overall smaller brain size, particularly in the hypothalamus, cerebellum, and hindbrain. Brain function was also compromised, with activity strongly increased in the cerebellum and abnormal behavior at baseline and in response to visual and acoustic stimuli. RNA-sequencing of developing larvae revealed significant downregulation of genes that mark olfactory sensory neurons, the lateral line, and cerebellar Purkinje neurons. Corroborating the RNA-sequencing, staining revealed fewer lateral line neuromasts and reduced Parvalbumin signal in the cerebellum. This study sets the stage for determining which downstream pathways underlie the emergence of the observed phenotypes and establishes multiple strong phenotypes that could form the basis of a drug screen.

## Introduction

Mutations in the gene early B cell factor 3 (*EBF3*) cause a neurodevelopmental disorder characterized mainly by intellectual disability and hypotonia. This genetic condition, known as Hypotonia Ataxia, and Delayed Development Syndrome (HADDS), was independently discovered in 2017 by 3 large sequencing projects, which together identified over twenty individuals (Chao *et al*. 2017; Harms *et al*. 2017; Sleven *et al*. 2017). Prior to the discovery of these coding variants, *EBF3* was hypothesized to contribute to intellectual phenotypes in individuals with terminal 10q deletions (Courtens *et al*. 2006; Faria *et al*. 2016), and many more cases have since been recognized (Blackburn *et al*. 2017; Tanaka *et al*. 2017; Jiménez de la Peña *et al*. 2021; Ciaccio *et al*. 2023). Sequence variation in *EBF3* includes heterozygous nonsense, splice, frameshift, and predicted deleterious missense mutations. While features of autism are present in only a subset of those with *EBF3* coding mutations, noncoding de novo variants in an *EBF3* enhancer were found in families with autism (Padhi *et al*. 2021). Other phenotypes found only in some individuals with *EBF3* mutations include motor coordination issues, ataxia, facial dysmorphism, pain insensitivity, ophthalmologic problems, short stature, restless sleep, and febrile seizures. Neuroimaging has uncovered cerebellar hypoplasia in some cases, although no other consistent structural abnormality has emerged (Harms *et al*. 2017; Tanaka *et al*. 2017; D’Arrigo *et al*. 2020).

Orthologs of *EBF3* are known to function in brain development in multiple species. While *Drosophila melanogaster* and *Caenorhabditis elegans* each only have a single Collier/Olf/EBF transcription factor, compared with 4 in most vertebrates, the protein has neurodevelopmental roles in both species (Crozatier *et al*. 1996; Prasad *et al*. 1998; Crozatier and Vincent 2008). In mice, homozygous disruption of *Ebf3* reduced survival, mating efficiency, and growth, and resulted in a failure of olfactory neurons to project to the olfactory bulb (Wang *et al*. 2004). Also in mice, *Ebf3* was shown to be a direct downstream target of the transcriptional repressor *Arx* (Fulp *et al*. 2008), which itself is involved in a neurodevelopmental syndrome consisting of intellectual disability, seizures, and dystonia (Strømme *et al*. 2002). In *Xenopus*, the *EBF3* ortholog regulates neuronal differentiation, inducing ectopic neurons when overexpressed in the developing embryo (Pozzoli *et al*. 2001). In most brain regions and at multiple stages of development, *ebf3* has a similar expression pattern to the *Xenopus* ortholog of mammalian *NEUROD1*, including the neural tube, neural crest cells, olfactory placodes, trigeminal placodes, spinal cord, and retina (Pozzoli *et al*. 2001; Green and Vetter 2011). Although *ebf3* was shown to be a direct downstream target of the NeuroD transcription factor, it also indirectly regulates *neurod1* expression, suggesting a more complex genetic interaction, possibly with feedback (Green and Vetter 2011).

Although *EBF3* orthologs have been studied in several species, a zebrafish mutant model has not been characterized. The genetically tractable zebrafish has proven useful for the study of the developmental and behavioral impacts of knocking out genes involved in neurodevelopmental disorders (Hoffman *et al*. 2016; Kozol *et al*. 2021; Zoodsma *et al*. 2022; Capps *et al*. 2024). Many features of zebrafish make it a powerful model to study these disorders, including optical transparency, external fertilization, and conservation of brain organization and many neuron types (Bae *et al*. 2009; Mueller 2012; Hashikawa *et al*. 2020; Porter and Mueller 2020; Pandey *et al*. 2023). Large-scale pharmacological screens for behavioral outputs or using reporter lines are also possible using larval zebrafish once a mutant phenotype has been established (Kokel *et al*. 2010; Rihel *et al*. 2010; Wang *et al*. 2015).

Here, we describe the characterization of the zebrafish orthologs of *EBF3*. In zebrafish larvae, expression of the *ebf3a* ortholog of *EBF3* was found in the posterior tuberculum area of the diencephalon, including in dopamine neurons, the midbrain-hindbrain boundary, olfactory bulb, neuromast hair cells, and hindbrain, including in cerebellar Purkinje cells (Li *et al*. 2010; Takeuchi *et al*. 2017; Lush *et al*. 2019). The *ebf3b* ortholog is minimally expressed (White *et al*. 2017; Bradford *et al*. 2022). Because of the *ebf3a* expression pattern and importance to brain development of other species, we expected numerous neural and behavioral phenotypes in mutants. Using brain imaging and larval behavioral profiling, we found changes in brain size, brain activity, baseline behavior, and stimulus-driven responses. As *EBF3* is a transcription factor, we also expected gene expression differences to likely drive the observed phenotypes. A microarray screen in *Xenopus* revealed a small number of direct and indirect *ebf3* target genes (Green and Vetter 2011), but RNA-sequencing datasets only exist for human cell lines (Harms *et al*. 2017), not for developing vertebrate embryos with manipulated levels of their *EBF3* ortholog. Thus, we collected RNA-sequencing data from our *ebf3a* zebrafish mutant, which nominated disrupted pathways and impacted cell types. Both cerebellar Purkinje cells and the lateral line were strongly affected by *ebf3a* loss. This study provides a starting point for further in-depth investigations into the *ebf3a* downstream target genes that drive these phenotypes and drug screens to discover molecules that ameliorate them.

## Materials and methods

### Zebrafish husbandry and mutant generation

Zebrafish (*Danio rerio*) were maintained in the Zebrafish Research Facility at the University of Alabama at Birmingham at 28°C on a 14-h light/10-h dark cycle. All animal studies were performed according to the guidelines by the Institutional Animal Care and Use Committee of the University of Alabama at Birmingham (IACUC protocols 22279, 22155, and 21744).

**Fig. 1.**
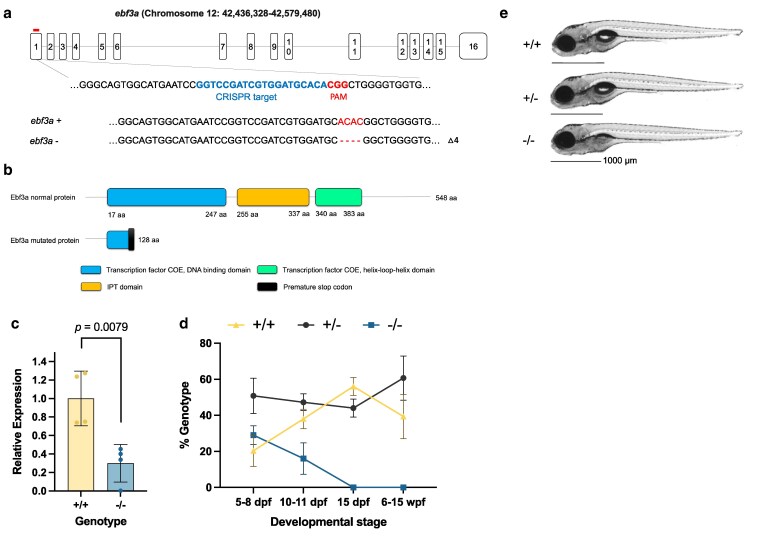
Generation and developmental characterization of zebrafish *ebf3a* mutants. a) Schematic of zebrafish *ebf3a* gene transcript and CRISPR gRNA targeting sequence. b) Schematic of zebrafish WT and mutant Ebf3a proteins. Domains are annotated based on ensemble Pfam database. c) Expression level of *ebf3a* in WT (+/+) and homozygous mutant (−/−) animals at 5 dpf, normalized to the WT level. d) Survival of *ebf3a* homozygous mutants (−/−) and respective siblings over larval development. e) Representative photos of *ebf3a* homozygous mutants (−/−) and respective siblings at 5 dpf.

*ebf3a* and *ebf3b* mutants were generated by microinjection of Cas9 protein and 1 guide RNA (gRNA) into AB wild-type (WT) zebrafish embryos as described previously (Wang *et al*. 2021). The target site, PAM motif, and identified out of frame mutant are listed in Fig. 1 and Supplementary Fig. 1. Experiments were completed using larvae derived from crosses using the F2 generation and later. To genotype mutants, genomic DNA was extracted from whole zebrafish embryos or tail clip from adult zebrafish using alkaline lysis. Then, High-Resolution Melting analysis was performed (Thomas *et al*. 2014): PCR with gene-specific primers (Supplementary Table 2) was completed in black/white 96 well plates (Bio-Rad cat. No. HSP9665) and followed by the generation of melting curves using a Lightscanner HR 96 (Idaho Technology). All experiments were conducted blind, and genotyping occurred after the data was collected.

### RNA extraction, real time quantitative PCR, and RNA-Seq

For RT-qPCR, total RNA was extracted from the anterior half of the body from 10 to 12 5 dpf larvae per sample at using the RNeasy Mini Kit (Qiagen). cDNA was synthesized using the High-Capacity cDNA Reverse Transcription Kit (Applied Biosystems). The RT-qPCR was performed using SsoAdvanced SYBR Green Supermix (Bio-Rad), primers (Supplementary Table 1), and CFX96 Touch Deep Well Real-Time PCR Detection System (Bio-Rad). RNA for the 2 dpf RNA-seq samples was collected in the same way, but with 15–20 embryos per pool. The 5 dpf RNA-seq was also collected with 10–12 larvae. The posterior portion of the cuts was used for genotyping. The RNA was isolated from the anterior portion as described and submitted to sequencing by GENEWIZ at Azenta Life Sciences. The number of input reads per sample ranged from 46 to 75 million, with uniquely mapped reads ranging from 35 to 57 million.

### RNA-sequencing analysis

Paired-end sequencing reads were aligned using STAR aligner (2.7.10a) (Dobin *et al*. 2013) to the GRCz11 release 104 using the Lawson Lab Zebrafish Transcriptome Annotation version 4.3.2 (Lawson *et al*. 2020). Using DESeq2 (Love *et al*. 2014), the raw counts files were normalized using rlog counts method. An example DESeq2 RScript and input files are available at https://github.com/thymelab/BulkRNASeq. The fold-change tables, all downstream analysis code, and complete results from transcriptome analyses are available in Supplementary Data 1 on Zenodo.

Gene Ontology (GO) analysis and Gene Set Enrichment Analysis (GSEA) (Subramanian *et al*. 2005) were performed using the clusterProfiler_4.10.0 R package, using the enrichGO and GSEA functions, respectively. To perform GSEA using the Daniocell single-cell data (Sur *et al*. 2023), the single-cell object was downloaded from the Daniocell website. Markers of the annotated clusters were identified using FindAllMarkers from Seurat_5.0.0 package (Satija *et al*. 2015), and the top 500 markers were used as gene sets for GSEA.

### Brain activity and morphology

Phosphorylated-ERK (pErk) antibody staining was conducted and analyzed as previously described (Thyme *et al*. 2019; Capps *et al*. 2024). Larvae were kept in quiet conditions for a minimum of a half hour before rapid fixation. They were fixed overnight at 4°C in 4% paraformaldehyde (Polysciences) diluted in 1X Phosphate Buffered Saline, permeabilized with 0.05% Trypsin-EDTA on ice for 30–35 min, stained for 2–3 days with the total ERK antibody (Cell Signaling, #4696, 3/1000 dilution) and phospho-Erk antibody (Cell Signaling #4370, 1/500 dilution), and 1–2 days with Alexa Fluor 647 mouse (A21235, 1/500 dilution) and 488 rabbit (A11008, 1/500 dilution) secondary antibodies (Thermo Fisher Scientific). After imaging with a Zeiss 900 upright confocal with 20× 1.0 NA water-dipping objective, larvae were removed from agarose for genotyping. As previously, the confocal stacks were registered to the Z-Brain atlas and processed using MapMAPPING with a false discovery rate of 0.05% (Randlett *et al*. 2015; Capps *et al*. 2024).

### Purkinje neuron staining

Purkinje cell antibody staining was conducted and analyzed as described above. Larvae were fixed at 6 dpf and stained for 2–3 days with total ERK antibody (Cell Signaling, #4695, 3/1000 dilution) and Anti-Parvalbumin Antibody (Sigma Aldrich, MAB1572, 1/500 dilution), and 1 day with Alexa Fluor 647 mouse (A21235, 1/500 dilution) and 488 rabbit (A11008, 1/500 dilution) secondary antibodies (Thermo Fisher Scientific). After imaging with a Zeiss 900 upright confocal with 20× 1.0 NA water-dipping objective, larvae were removed from agarose for genotyping. The analysis was completed similarly to above with MapMAPPING, but the Parvalbumin stain was not normalized by the total ERK stain, although the results were similar with both approaches.

### Lateral line staining

Neuromast staining was conducted as previously described (López–Schier *et al*. 2004; López-Schier and Hudspeth 2005). Live 6 dpf larvae were immersed for 15 min in a 250 μM solution of 4-(4-(diethylamino)styryl)-N-methylpyridinium iodide (4-Di-2-ASP: Millipore Sigma, D3418) at room temperature in the dark. Larvae were then rinsed to remove excess fluorophore, anesthetized with Tricain MS-222, and mounted in 2% LMP agarose. Larvae were removed from agarose after imaging for genotyping. The fluorescent neuromasts of each image were counted, while blinded to genotype, noting the total number of neuromasts and the number of neuromasts on the posterior lateral line (Manuel *et al*. 2021). While counting, mirrored neuromasts on the other side of the body were omitted to prevent double counting.

### Larval behavior

Larval behavior assays were completed using custom-built behavioral systems as previously described (Thyme *et al*. 2019; Joo *et al*. 2020). Briefly, on the evening of 4 dpf, the animals are placed in 96-well plates with square wells and sealed with oxygen permeable film. The white light cycle is 9 AM on/11 PM off. The larvae are exposed to the following stimuli at 5 dpf: light flashes (9:11–9:25), mixed acoustic stimuli (prepulse, strong, and weak, from 9:38 to 2:59), 3 blocks of acoustic habituation (3:35–6:35). The night between 5 and 6 dpf: mixed acoustic stimuli (1:02–5:00), light flashes (6:01–6:20). During the day at 6 dpf: 3 blocks of dark flashes with an hour between them (10:00–3:00), mixed acoustic stimuli and light flashes (4:02–6:00). The baseline framerate is 30 frames-per-second, while the responses to stimuli are analyzed from high-speed (285 frames-per-second) videos of a 1-s duration. All code for behavior analysis is available at https://github.com/thymelab/ZebrafishBehavior. As previously, the significance of data from baseline blocks, such as the duration of the experiment, are calculated with a linear mixed model (Thyme *et al*. 2019; Joo *et al*. 2020), while stimulus response data is assessed using the more standard Kruskal–Wallis ANOVA. The ANOVA values for these longer durations, with large variation in the measures across time, are also available in the supplementary files on Zenodo. To generate the bubble plot summary visualization, related measures are combined. The size of the bubble represents the percent of significant measurements in the summarized category, and the color represents the mean of the strictly standardized mean difference (SSMD) of the significant assays in that category. The bubbles are offset and both included (vs a typical heatmap), because both decreased and increased measures can occur in the same summarized block, such as if a change occurs between the 4 and 6 dpf stages. Another potential example is if the bout velocity is increased but the distance traveled in bouts is decreased, as both are bout “magnitude” measures. The scripts for merging these measurements and generating the summarized bubble plot graphs are available at https://github.com/thymelab/DownstreamAnalysis.

## Results

### Zebrafish *ebf3a* is the functional ortholog of human *EBF3*

There are 2 putative orthologs of human *EBF3* in the zebrafish genome: *ebf3a* and *ebf3b*. A common isoform of the Ebf3a protein (548 aa) has 88.8% identity with human EBF3 (596 aa), while Ebf3b (514 aa) has 62.7% identity. To investigate the function of these orthologs, we used CRISPR/Cas9 to establish loss-of-function mutant lines for *ebf3a* and *ebf3b* (Fig. 1a, Supplementary Fig. 1a). Both mutations are predicted to cause frameshift of the genes and a premature stop codon, leading to protein truncation at amino acids 128 and 124 for Ebf3a and Ebf3b, respectively (Fig. 1b, Supplementary Fig. 1b). Using RT-qPCR, we confirmed a significant reduction of the *ebf3a* transcript in the corresponding mutant (Fig. 1c). For *ebf3b*, Quantification Cycle (Cq) values were consistently over 30 in WT and homozygous samples, indicating a low mRNA level (data not shown). This result is corroborated by published bulk RNA-seq data (White *et al*. 2017; Bradford *et al*. 2022) and recent single-cell RNA-seq atlases (Sur *et al*. 2023; Pandey *et al*. 2023) (Supplementary Fig. 1c–e). Single-cell RNA-sequencing revealed high expression of *ebf3a* in neurons across several tissue types (brain, eye, olfactory, and lateral line) and almost no expression of *ebf3b* in the whole organism from 0 to 5 days post-fertilization (dpf) (Supplementary Fig. 1c). Its lower expression level and protein identity indicate that *ebf3b* may not have retained function following zebrafish genome duplication.

First, we evaluated the morphology and survival of these 2 mutants. We failed to detect adult *ebf3a* F2 homozygous mutants and determined that lethality occurred as early as 10 dpf (Fig. 1d, Supplementary Table 1). At larval stages, we found that the swim bladders of *ebf3a* homozygous mutants were not inflating (Fig. 1e), while the *ebf3b* larvae were normal (Supplementary Fig. 1f). In 1 clutch, the absent swim bladder was observed in 20 out of 35 homozygous larvae at 6 dpf and only 3 out of 133 heterozygous and WT siblings. In summary, we generated an *ebf3a* mutant model with developmental phenotypes, further supporting the designation of *ebf3a* as the functional ortholog at early developmental stages.

### Zebrafish *ebf3a* mutants have altered brain morphology, brain activity, and behavior

**Fig. 2.**
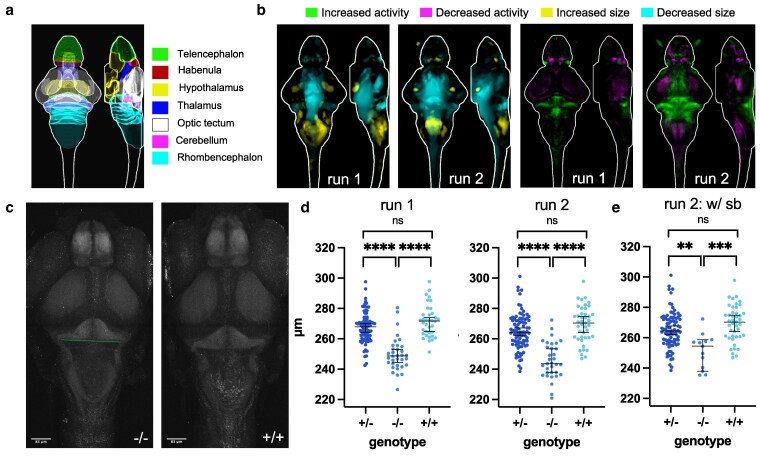
Brain structure and activity phenotypes of zebrafish *ebf3a* mutants. a) Schematic of major zebrafish brain regions based on the Z-Brain atlas (Randlett *et al*. 2015). b) Brain structure and activity maps for 2 runs from separate clutches, comparing homozygous mutants (−/−) to WT (+/+) siblings. A sum-of-slices intensity projection is shown (Z- and X- axes) of the signals inside the brain (white outline). Run 1 *N* = 34 homozygotes (−/−) compared with 36 wild types (+/+); Run 2 *N* = 36 homozygotes (−/−) compared with 46 wild types (+/+). c) Example of measuring the width of the hindbrain in the cerebellum region on the raw confocal stacks. The line represents the measurement that was made on maximum-intensity projections of the tErk stain. d) Measurement of the hindbrain width in all animals from the 2 runs. The *P*-values for both runs are <0.0001 for comparisons of homozygous mutants (−/−) to both WT (+/+) and heterozygous (+/−) siblings. *P*-values were calculated using the Brown-Forsythe and Welch ANOVA with Dunnett's T3 multiple comparisons test (GraphPad Prism 10). e) Measurement of the hindbrain width for only those with swim bladders from run 2. The *P*-values are 0.0002 for homozygous mutants (−/−) compared with WT (+/+) and 0.0024 for homozygotes (−/−) compared with heterozygotes (+/−).

Given the known role of its orthologs in the nervous system and neural expression, we next assessed the *ebf3a* mutant using pErk brain activity mapping (Randlett *et al*. 2015; Thyme *et al*. 2019). In this approach, 6 dpf larvae are stained for pErk and total-Erk (tErk), image stacks registered to the Z-Brain atlas (Randlett *et al*. 2015), and the differences in pErk/tErk ratio between mutant and sibling groups are a proxy for activity. In addition, the registration process generates a deformation matrix that can be similarly compared with identify brain areas (Fig. 2a) with increased or decreased size (Jefferis *et al*. 2007; Thyme *et al*. 2019). The *ebf3a* homozygous mutants showed altered brain activity and structure compared with their WT siblings, with replicable outcomes from 2 separate clutches (Fig. 2b). The size of the brain was generally smaller, particularly in the hypothalamus, cerebellum, and hindbrain, although an area of the caudal rhombencephalon was increased in size. Increased brain activity was pronounced in the cerebellum, as well as in the optic tectum neuropil in the run with stronger signal, and reduced activity was observed in the telencephalon, habenula, mesencephalon, and caudal rhombencephalon. There were differences in both morphology and activity in the olfactory bulb and eye (Supplementary Fig. 2a–c). Examination of the raw image stacks confirmed the smaller size of the cerebellum (Fig. 2c and d). To ensure that these phenotypes were not caused by the lack of a swim bladder, we separately analyzed only those where it had fully inflated (Fig. 2e, Supplementary Fig. 2d) Although the phenotype was milder, as would be expected from the smaller N and selection of least impacted animals, the reduced cerebellar size and increased caudal rhombencephalon remained, as did the increased activity in the cerebellum and reduced in the habenula. Although these strong phenotypes were absent in heterozygous siblings, mild, repeatable activity differences in the cerebellum and habenula remained (Supplementary Fig. 2e).

**Fig. 3.**
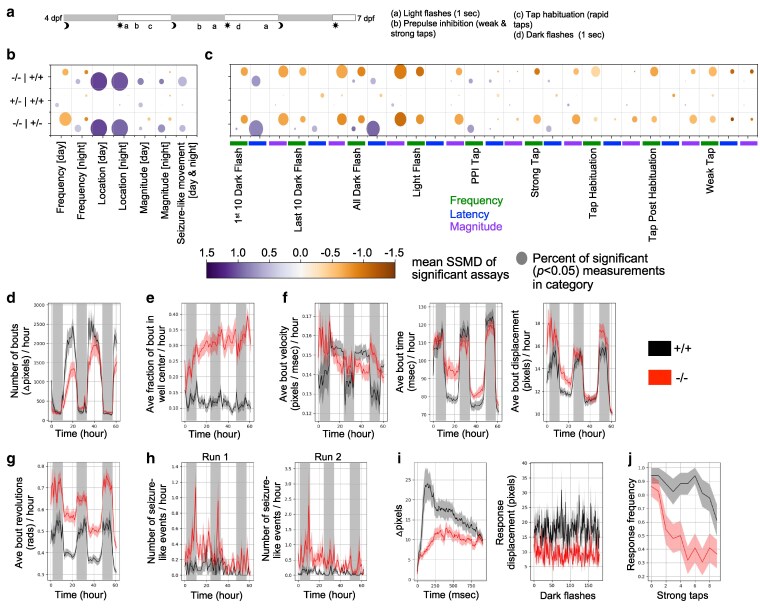
Behavioral phenotypes of zebrafish *ebf3a* mutants. a) Multiday behavioral pipeline with acoustic and visual stimulation. b) Summary visualization of baseline behavioral phenotypes in *ebf3a* homozygous mutants (−/−) compared with respective siblings. The size of the bubble represents the percent of significant measurements in the summarized category, and the color represents the mean of the SSMD of the significant assays in that category. Run 1 *N* = 23 homozygotes (−/−), 18 wild types (+/+), and 36 heterozygotes (+/+). c) Summary visualization of stimulus-driven behavioral phenotypes in *ebf3a* homozygous mutants compared with respective siblings. d) Example of a frequency of movement phenotype, shown for the entire duration of the experiment. This measure is the number of bouts, binned per hour, calculated using the change in pixels between each frame (linear mixed model *P*-value = 0.005). e) Example of a location preference plot, shown for the direction of the experiment. This measure is the fraction of the frames of a bout that is spent in the center zone of the well, binned per hour, calculated based on the centroid positions of the fish (*P*-value = 0.001). f) Examples of bout magnitude measures, shown for the duration of the experiment, binned per hour, and calculated based on the centroid positions of the fish. The bout velocity is not significant when considering the experiment duration, but subregions do have significant *P*-values (e.g. the third night or day3nightall, binned per 10-min, *P*-value = 0.001). Bout time *P*-value for the experiment duration = 0.025, and bout displacement = 0.001. g) Bout revolutions for the duration of the experiment (*P*-value = 0.001). h) Movements that resemble seizures (*P*-value = 0.001 for both sets). These are calculated based on the movement including more than 4 full revolutions, moving a distance of over 70 pixels, and having a speed of 0.3–1.3 pixels/msec. i) Displacement for the dark flash response movement (right). Kruskal–Wallis ANOVA *P*-value = 1.7e−06. The response graph (right) is the average movement (pixel-based) of larvae in the homozygous mutant and WT control groups for events where a response was observed. j) Frequency of responses to strong taps. The block shown is the strong taps completed prior to the first habituation block at 5 dpf (day5dpfhab1pre), with Kruskal–Wallis ANOVA *P*-value = 9.2e−05. Plots of homozygous mutant (−/−) compared with WT (+/+) control groups in panels (d–j) are mean ± SEM.

As they displayed significant changes to brain structure and function, we expected these larvae to have behavioral phenotypes. Thus, we assessed them using a multiday battery that included visual and acoustic stimulation (Fig. 3a). Homozygous mutants displayed repeatable differences in baseline and stimulus-driven measures compared with control WT siblings (Fig. 3b and c). Two runs from biologically distinct clutches corroborated this result (Supplementary Fig. 3a–d). While mutants tended to have fewer daytime bouts compared with WT larvae (Fig. 3d, Supplementary Fig. 3e), stronger phenotypes were observed in their location preference (Fig. 3e, Supplementary Fig. 3f) and structure or magnitude of these bouts (Fig. 3f, Supplementary Fig. 3h). They preferred the center of the well, and the individual movements had altered velocity, time, and displacement, which are all related measures. These animals had a marked increase in the number of revolutions they made in the bout (Fig. 3f, Supplementary Fig. 3g), a measure that is closely linked to their increased number of seizure-like movements (Fig. 3h, Supplementary Fig. 3i), quantified based on high-speed circling behavior (Baraban *et al*. 2005). When exposed to visual or acoustic stimuli, the mutants had reduced responses (Fig. 3i and j, Supplementary Fig. 3j and k). The magnitude of the dark flash response was particularly impacted.

### Transcriptional differences in zebrafish *ebf3a* mutants indicate impacted development of multiple sensory systems and the cerebellum

**Fig. 4.**
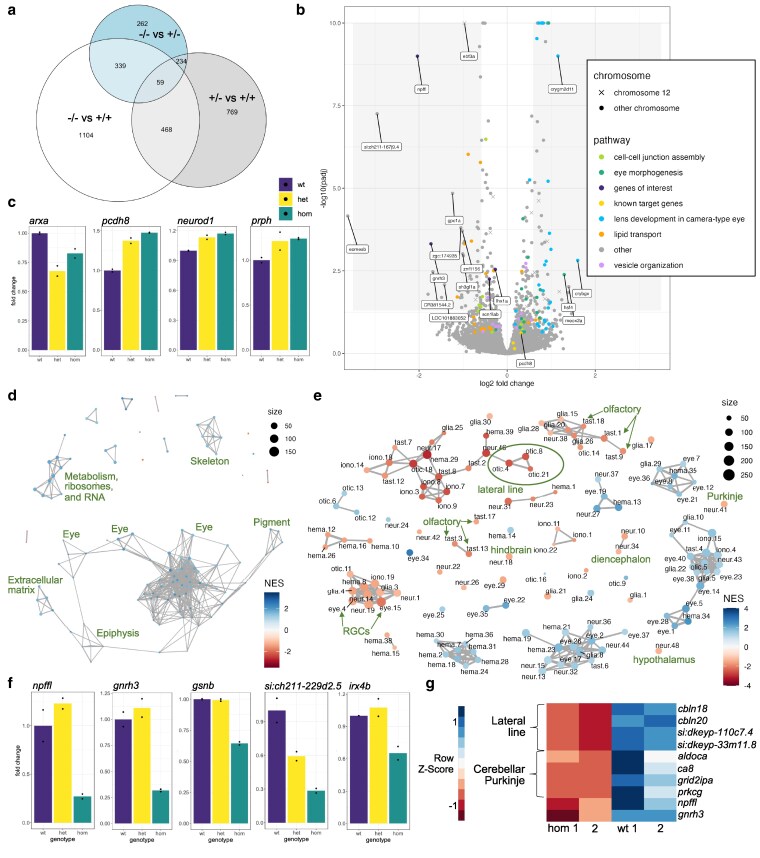
Transcriptional changes in zebrafish *ebf3a* mutants. a) Venn diagram of shared DEGs between the genotype comparisons of 2 dpf RNA-Sequencing data. The DEGs were filtered using a *P*-value of <0.05 and log2 fold change of >0.2. b) Volcano plot of the 2 dpf RNA-Sequencing data for the comparison of homozygous mutants vs combined heterozygous and WT larvae. Genes involved in pathways identified by GO analysis are identified. Genes with log2 fold changes of >1.0 and adjusted *P*-values of <0.01 are labeled, as are several additional genes of interest. c) Normalized counts data for WT, heterozygous, and homozygous samples for genes selected based on known involvement with *ebf3*. d) Network plot of all GSEA C5 molecular signatures for the comparison of 2 dpf homozygous mutants vs combined heterozygous and WT larvae, with general groupings labeled. e) GSEA network plot with single-cell types from the Daniocell larval zebrafish atlas. Clusters of particular interest are labeled with arrows and a circle to designate the clusters corresponding to the lateral line. f) Normalized counts data for WT, heterozygous, and homozygous samples for genes with strong downregulation in *ebf3a* mutants and mark specific neuron types. g). Genes that are downregulated in 5 dpf RNA-Sequencing data and mark specific neuron types. All analysis code, additional results, terms and genes found by the GO and GSEA analyses, and necessary files for the panels of this figure are available in Supplementary Data 1 in the Zenodo repository.

To define the molecular basis for the observed neural phenotypes, we collected bulk RNA-sequencing data from *ebf3a* mutants at 2 dpf. This age was selected to uncover early molecular changes that could drive later phenotypes. Multiple differentially expressed genes were identified in all genotype comparisons (Fig. 4a). As expected, *ebf3a* itself was significantly downregulated (Fig. 4b). Many genes involved in eye development, which we grouped using GO analysis, were upregulated in the homozygous mutants compared with combined data from WT and heterozygous siblings as well as WT siblings alone (Fig. 4b, Supplementary Fig. 4a, Supplementary Data 1). We compared homozygous to the combined data to highlight the transcriptional changes most indicative of brain morphology phenotypes, which were not present in heterozygous or WT siblings. Other categories of dysregulated genes included lipid transport, cell-cell junction assembly, and vesicle organization. Surprisingly, no terms related to neuronal development emerged from GO analysis when the homozygous samples were compared with the combined data (Fig. 4b), and only a small number of genes related to axon development were identified in the homozygous vs WT analysis (Supplementary Fig. 4a). Among the genes with significantly reduced expression in both comparisons, however, were some with known involvement in brain development, such as *lhx1a* and *scn1lab* (Swanhart *et al*. 2010; Baraban *et al*. 2013; Lui *et al*. 2017; Symmank and Zimmer-Bensch 2019; Mendes *et al*. 2023). Among the most reduced genes in homozygous samples was *gpc1a*, the loss of which leads to reduced brain size in mice, possibly via reduced fibroblast growth factor signaling (Jen *et al*. 2009). In zebrafish, *gpc1a* is expressed widely in the central nervous system, as well as also in the lateral line and some olfactory sensory neuron subtypes (Gupta and Brand 2013; Sur *et al*. 2023). Both the known Ebf3 target genes *pcdh8*, *neurod1*, and *prph* and the *ebf3* upstream repressor *arxa* were also differentially expressed (Fulp *et al*. 2008; Green and Vetter 2011) (Fig. 4c). The *arxa* transcript was downregulated, and there is a predicted binding site for Ebf3a in the *arxa* promoter as well as a predicted binding site for Arxa in the *ebf3a* promoter (Supplementary Fig. 5) (Rauluseviciute *et al*. 2024). However, the known target genes were unexpectedly upregulated in heterozygous and homozygous samples, opposite to the results from microinjection of mRNA and morpholinos in *Xenopus* (Green and Vetter 2011).

Using GSEA, we highlighted additional pathways disrupted in the *ebf3a* mutants. For the homozygous mutants (Fig. 4d, Supplementary Fig. 4b), enrichment using the C5 set of molecular signatures overlapped with the GO analysis, with most terms resulting from the upregulation of eye genes. In the heterozygous samples compared with the WT; however, many more genes involved in neuronal development, circadian behavior, posterior lateral line system development, channel activity, ion transport, and synapse assembly were uncovered by both GSEA and GO (Supplementary Fig. 6, a and b). The differentially expressed genes in the heterozygous data overall had smaller fold changes than in the homozygous. This comparison, however, may reflect target genes of *ebf3a* better than the homozygous data that could be confounded by signatures of their altered development.

To predict impacted cell types, we also used GSEA to identify marker genes of cell types derived from scRNA-seq of the developing larvae (Sur *et al*. 2023). Genes selectively expressed in neuron types from multiple sensory systems were downregulated, including olfactory, visual retinal ganglion cells (RGCs), and lateral line subtypes (Fig. 4e, Supplementary Fig. 4c). Ebf3a is known to be expressed in RGCs, acting downstream of Atoh7 (Gao *et al*. 2014). The diencephalon, including the hypothalamus, and hindbrain, including the cerebellar Purkinje cells, emerged from the analysis of homozygous mutants compared with the combined data from heterozygotes and wild types. Highly specific marker genes of these nominated clusters were downregulated, such as olfactory sensory neurons (Supplementary Fig. 7a). Two of the most differentially expressed genes, *npffl* and *gnrh3* (Fig. 4f) are in so few cells that they are not obviously linked to specific neuron types in Daniocell, but their expression correlates with each other (Sur *et al*. 2023), and *gnrh3* is selectively expressed in cells near the olfactory placodes in larvae (Kurrasch *et al*. 2009; Bassi *et al*. 2020). Although the strongly reduced gene *gsnb* (Fig. 4f) is expressed in multiple cell types, including the lateral line and olfactory system, other more specific markers such as *si:ch211-229d2.5* are expressed only in the lateral line and no other cell type (Supplementary Fig. 7b), supporting the GSEA results (Fig. 4e). Although cerebellar Purkinje cells are not detectable by Parvalbumin staining until 3 dpf (Kani *et al*. 2010), multiple transcriptional markers of this type are downregulated in our 2 dpf data, including *irx4b*, *ppargc1a*, and *rnf152* (Fig. 4f, Supplementary Fig. 7c). The transcription factor *ptf1a*, which marks the progenitors that give rise to Purkinje cells (Kani *et al*. 2010), was not differentially expressed (Supplementary Data 1). Most differentially expressed genes from RNA-seq collected at the later stage of 5 dpf were also highly specific markers of the lateral line and Purkinje cells (Fig. 4g, Supplementary Fig. 8), although the samples were less consistent, and no heterozygous were included. Despite the different developmental stage and lower quality of the 5 dpf data, multiple genes were shared between the 2 datasets (Supplementary Fig. 8). Taken together, while molecular signatures of aberrant neuronal development were only prominent in the heterozygous samples, a comparison to scRNA-seq marker genes revealed that multiple sensory systems and specific brain areas were predicted to have fewer or missing differentiated neurons in the homozygous larvae.

**Fig. 5.**
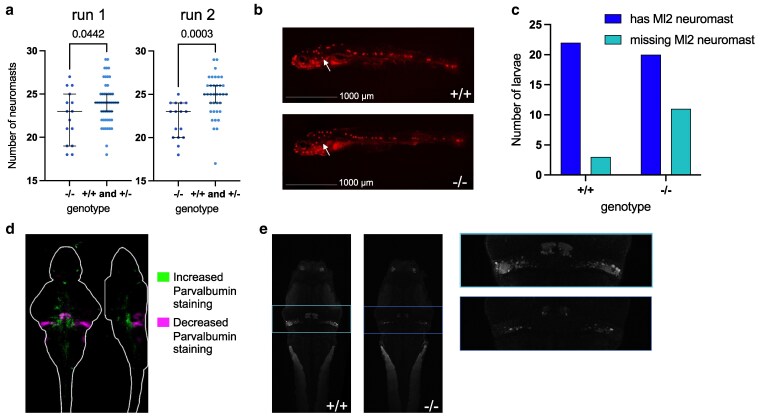
Staining of the lateral line and Purkinje neurons in zebrafish *ebf3a* mutants. a) Counts for lateral line neuromasts on one side of the 6 dpf zebrafish. Run 1 *N* = 15 −/− and 50 +/− and +/+; Run 2 *N* = 16 −/− and 36 +/− and +/+. Significance was assessed using an unpaired *t*-test. b) Representative image of lateral line staining. The white arrow indicates the MI2 neuromast which was absent in some homozygous mutant larvae. c) Combined quantification of missing MI2 neuromasts from both runs. Only homozygous mutants and WT siblings were included. One-sided Chi-square test: *P* = 0.0218. d) Brain map of the quantified differences in Parvalbumin staining, comparing homozygous mutants (−/−) to WT (+/+) siblings. A sum-of-slices intensity projection is shown (Z- and X- axes) of the signals inside the brain (white outline). *N* = 14 homozygotes (−/−) compared with 26 wild types (+/+). e) Example slice of Parvalbumin staining from registered stacks. The slice is at position 100 out of 138 in the standard Z-Brain stack. The cerebellum region is boxed and shown enlarged to the right.

To validate the RNA-sequencing predictions, we stained lateral line neuromasts and cerebellar Purkinje neurons at 6 dpf. Although neuromasts were still present in homozygous mutants, their numbers were significantly reduced (Fig. 5a, Supplementary Fig. 9a). In particular, we noted that the MI2 neuromast was more frequently absent in homozygotes (Fig. 5b, c). To assess cerebellar neurons, we stained larvae for Parvalbumin, which is expressed in Purkinje cells. Using a protocol similar to that used for pErk-based brain activity mapping, image stacks were registered and compared, revealing a significant reduction in cerebellar signal (Fig. 5d). While this antibody also labels neurons in other regions of the brain, the cerebellar signal was especially diminished (Fig. 5e, Supplementary Fig. 9b). These findings corroborate the RNA-sequencing results and support a disruption of both sensory and motor-related neuronal populations in the homozygous mutants.

## Discussion

Here, we describe the characterization of an *ebf3a* zebrafish mutant. Loss of this *EBF3* ortholog results in substantial changes to brain structure, brain activity, and behavior, as well as lethality at late larval stages. Using GSEA in combination with single-cell marker genes highlighted that the lateral line and cerebellar Purkinje cells (Fig. 4) are possibly missing or fewer in number. Sequencing data from both 2 and 5 dpf support this conclusion, which was corroborated with staining for both populations (Fig. 5). Our results align well with one another, as the imaging (Fig. 2) indicated a smaller cerebellum, and the altered movement structure (Fig. 3) could reflect the impacted cerebellum and hindbrain. While our finding might have been expected, as Purkinje cell numbers are markedly decreased in mouse *Ebf2* null mutants (Croci *et al*. 2006) and *ebf3a* is expressed in this cell type, no specific data existed on orthologs of *EBF3* and cerebellar development. Our study also highlights other less obvious neuron types for future exploration, such as a hypothalamus subtype (neur.48) that is specifically marked by *lmx1al* (Sur *et al*. 2023).

Some zebrafish phenotypes resemble those found in individuals with *EBF3* mutations. Ataxia and challenges with motor coordination are common. While it is not generally known how a larval zebrafish with ataxia would behave, we did observe changes in the bout structure (Fig. 3f). Responses to stimuli were also dampened (Fig. 3i and j), possibly due to abnormal processing of the sensory input or motor coordination of the response. The cerebellum, particularly Purkinje cells, is strongly implicated in many ataxias (Huang and Verbeek 2019). A consistent finding in neuroimaging data is cerebellar hypoplasia (Harms *et al*. 2017; Tanaka *et al*. 2017; D’Arrigo *et al*. 2020), which we also observed in the homozygous mutants (Fig. 2b and f). The reduced response to dark flashes (Fig. 3i) and dysregulation of genes involved in eye development (Fig. 4b and d) could arise similarly or contribute to ophthalmologic problems present in individuals with HADDS such as strabismus and hypermetropia (Harms *et al*. 2017; Lopes *et al*. 2017). The zebrafish larvae also displayed seizure-like movements, and abnormal EEG results and febrile seizures did occur in some individuals carrying damaging mutations in *EBF3* (Sleven *et al*. 2017).

One limitation of our model is that individuals with *EBF3* mutations maintain 1 WT copy of the gene whereas the phenotypic zebrafish are homozygous mutants. Human mutations include frameshifts, gene deletions, and missense alleles, which can reduce transcriptional output in cell-based assays through haploinsufficiency or a dominant negative mechanism (Chao *et al*. 2017; Harms *et al*. 2017; Lopes *et al.* 2017; Sleven *et al*. 2017). From our lethality data, we expect that a complete loss of *EBF3* would also not be compatible with human survival. The necessity of homozygosity to yield strong phenotypes in zebrafish with protein-truncating mutations in neurodevelopmental disorder genes is a common phenomenon (Hoffman *et al*. 2016; Zoodsma *et al*. 2022; Mendes *et al*. 2023; Capps *et al*. 2024). It is likely that genetic compensation mediated by the premature stop codon is responsible for the limited heterozygous phenotypes, as it could result in upregulation of the *ebf3a* WT allele or other transcription factors with a similar sequence that could act in its place (El–Brolosy *et al*. 2019). While the *ebf3a* level is reduced in the heterozygous samples (Supplementary Fig. 6a), rather than upregulated as we have seen previously (Capps *et al*. 2024), its change is far less than then expected 50% (Supplementary Data 1: −0.14 log2 fold change, *P*-value = 0.016). We did not observe an upregulation of *ebf3b* or *ebf2*, but *ebf1a* and *ebf1b* were slightly upregulated. In the future, making a new mutant line with the promoter removed, which would not produce truncated RNAs that drive this compensation, could yield stronger heterozygous phenotypes. It is also possible that this compensation could come through genetic mechanisms other than nonsense-mediated decay. The *arxa* gene is downregulated in both heterozygous and homozygous samples (Fig. 4c), and *Ebf3* was shown to be a direct target of *Arx* in the developing mouse brain (Fulp *et al*. 2008). When *Arxa* is reduced, *Ebf3* is upregulated, and it is possible that *arxa* is also a target of *ebf3a* and is itself downregulated to derepress *ebf3a*. Each transcription factor has a predicted corresponding binding site in the others promoter (Supplementary Fig. 5), suggesting complex but direct cross-regulation.

Although the heterozygous mutants had very mild phenotypes (Fig. 2g), their transcriptional dysregulation was extensive (Supplementary Fig. 6). The levels of target genes known from the literature (Fig. 4c) are as strongly increased in heterozygous and homozygous, and the *arxa* was downregulated in both. While the expectation is that the level of target genes would be reduced in correspondence to lower *ebf3a*, those experiments were done using transient microinjection in *Xenopus* (Green and Vetter 2011), which could have a different outcome than for our germline mutant. Pathway analysis uncovered gene sets involved in brain structure and function, which was not the case for homozygous mutants. Sets of interest included circadian behavior, as individuals with *EBF3* mutations have disturbed sleep, and multiple ion channel subunits. While it is surprising that there is significant transcriptional dysregulation with mild observed neural phenotypes (Fig. 2g), these gene sets could more directly reflect the transcriptional response to reducing *ebf3a*, rather than the emergent morphological changes in mutants. There are likely missing or disproportionately skewed cell populations in the homozygous mutants and not in the heterozygous siblings. A goal of our work was to uncover downstream target genes of *ebf3a* that could be responsible for the mutant neural phenotypes, and those dysregulated in heterozygous samples and more so in homozygous could be promising for further study.

Several strongly dysregulated genes are possible drivers of the observed phenotypes. Zebrafish mutants in *scn1lab*, which is downregulated in *ebf3a* homozygous mutants, have reduced size in the midbrain-hindbrain boundary region (Mendes *et al*. 2023). This region overlaps with the area that is ventral to the cerebellum and smaller in *ebf3a* mutants (Fig. 2b). Brain activity differs between the 2, however, as *scn1ab* has reduced activity throughout the entire brain compared with the strong activity increase in the cerebellum of *ebf3a* mutants. Behaviorally, both display seizure-like movements (Baraban *et al*. 2013) (Fig. 3h), as do *arxa* mutants (Griffin *et al*. 2021). In the Purkinje cell cluster, *irx4b* is expressed before other marker genes (Supplementary Fig. 7c), beginning at 24–34 hpf in Daniocell when the cells are just beginning to be classified. This transcription factor is a possible early driver of Purkinje cell differentiation and has been linked to axonal pathfinding in retina (Jin *et al*. 2003). The progenitors that give rise to Purkinje cells (Kani *et al*. 2010) do not appear affected, as *ptf1a* is not differentially expressed. The genes *skor1a* and *skor1b*, which are key regulators of the first step in Purkinje cell differentiation from *ptf1a* progenitors, are also not differentially expressed. Collecting RNA-sequencing data from an earlier stage, such as 36 hpf, could support this hypothesis and reveal other early *ebf3a* target genes.

The morphological and behavioral phenotypes of *ebf3a* homozygous mutants could be starting points for drug discovery. Transgenic lines that specifically label the lateral line and Purkinje cells (Tanabe *et al*. 2010; Fuentes *et al*. 2016) could be used for an imaging-based drug screen (Wang *et al*. 2015). Seizure-like movements in zebrafish larvae, which we observe in *ebf3a* mutants, were used as a screening platform to find a new treatment for Dravet syndrome that is in clinical trials (Baraban *et al*. 2013; Dinday and Baraban 2015). Overall, our findings provide a foundation for understanding the role of *ebf3a* in neurodevelopment and offer a promising avenue for developing targeted therapeutic interventions.

## Supplementary Material

jkaf115_Supplementary_Data

## Data Availability

All data are available in the main text, the Supplementary Materials, or appropriate databases. Code is available in Supplementary Data 1 or from https://github.com/thymelab (specific repositories are noted in the methods: BulkRNASeq, ZebrafishBehavior, and DownstreamAnalysis). Supplementary Data 1 and processed behavioral and imaging data are available from Zenodo (10.5281/zenodo.15304810) (Thyme 2025). Additional RNA-seq analysis results and the analysis workflow are in Supplementary Data 1 on Zenodo. A description of the content of the files in the Zenodo Repository is available in the Supplementary Materials. Raw behavioral and imaging files that were too large for Zenodo are available upon request. RNA-sequencing data is available from GEO (GSE276705) (Thyme 2024). The *ebf3a*^uab447^ allele, ZFIN ID ZDB-ALT-221114-33, is available from ZIRC. Supplemental material available at G3 online.
