# Supplementary material for: Disrupted development of sensory systems and the cerebellum in a zebrafish ebf3a mutant
Source: G3 (Bethesda). 2025 May 23;15(7):jkaf115. doi: 10.1093/g3journal/jkaf115 (PMC12239631; doi:10.1093/g3journal/jkaf115)
Supplement: jkaf115_Supplementary_Data [file jkaf115_supplementary_data.zip › Supplemental_Material_G3-2025-405899.pdf]

List of files in Zenodo repository (10.5281/zenodo.15304810)

1. ebf3a-pvalb-nonorm_2025-04-08_Parvalbumin_14homover22het_hetfxhetm_p0005.tif
2. ebf3a-pvalb-nonorm_2025-04-08_Parvalbumin_14homover26wt_hetfxhetm_p0005.tif
3. ebf3a-pvalb-nonorm_2025-04-08_Parvalbumin_14homover48hetandwt_hetfxhetm_p0005.tif
4. ebf3a-pvalb-nonorm_2025-04-08_Parvalbumin_22hetover26wt_hetfxhetm_p0005.tif
5. behavior_ebf3a_Box12_11-20-2020_flo.tgz
6. behavior_ebf3a_Box2_01-18-2021_flo.tgz
7. behavior_ebf3a_Box4_03-19-2021_flo.tgz
8. genotyping_10-07-21set
9. genotyping_8-24-21set
10. genotyping_pvalb-stain-2025-04-08
11. images__ebf3a_8-24-21_CC.zip
12. images__ebf3a_het_jmp11_2021-10-07_CC.zip
13. Lateral_Line_Staining.zip
14. processed_imaging.zip
15. pvalbstaining_registeredimages.tgz
16. pvalbstaining_unregisteredimages.tgz
17. Supplementary Data S1.zip

Legends

Files 1-4: Differences in Parvalbumin staining between *ebf3a* mutants and control siblings. The genotype comparison and the N for each genotype is in the name of the files. Stacks resulting from MapMAPPING comparing the Parvalbumin stained fish. The Parvalbumin stain was not normalized to the total-Erk stain used for registration, but the results are similar when it is normalized (data not shown).

Files 5-7: Complete behavior data and analyzed files for three behavioral runs comparing *ebf3a* mutants and control siblings. The only raw data not included, due to size limitations, are the high-speed movie files, but their tracking data is included. The genotyping information is located within the individual archive.

Files 8-10: Genotyping information for the three imaging runs. The date in the name matches the date in the archive of raw imaging data.

Files 11 and 12: Raw confocal stacks stained with total Erk and phospho-Erk for *ebf3a* mutants and control siblings.

File 13: Raw images of lateral line staining comparing *ebf3a* mutants and control siblings. The samples are divided by genotype within the archive.

File 14: The results of brain activity and structural MapMAPPING for the total Erk and phospho-Erk staining. These processed datasets correspond to the raw stacks in Files 11 and 12.

Files 15 and 16: Raw Parvalbumin stained stacks: the original “unregistered” stacks (File 16) and the stacks after registration (File 15). The corresponding genotyping can be found in File 10.

File 17: Additional files related to the RNA-seq analysis, including the R Markdown file, differentially expressed gene lists, and GSEA terms based on Daniocell clusters.
